# Microbiota of *Crassostrea virginica* larvae during a hatchery crash and under normal production: Amplicon sequence data

**DOI:** 10.1016/j.dib.2021.107755

**Published:** 2021-12-23

**Authors:** Jacob A. Cram, Matthew W. Gray, Katherine McFarland, Ashley Hollins

**Affiliations:** a University of Maryland Center for Environmental Science, Horn Point Laboratory, Cambridge MD, United States; b NOAA Fisheries Service, Northeast Fisheries Science Center, Milford CT, United States

**Keywords:** Oyster larval microbiota, Amplicon sequences, DADA2 pipeline, Hatchery crash

## Abstract

We present oyster larval microbiota from two feeding studies, in which wild type and low-salinity tolerant lines were either fed or starved. In one study, all larvae unexpectedly died, which was concurrent with an event in which all larvae in an adjoining oyster hatchery also died. In the other study, no crash occurred in either the study or hatchery. In both cases, larvae were collected and stored frozen, and microbial and host DNA was isolated by phenol-chloroform extraction. Both host 18 s rRNA genes and microbial 16 and 18 s rRNA genes were sequenced using universal primers. We present raw sequences, the pipeline that was used to quantify amplicon sequence variants, and our analysis pipeline that we used to describe how the overall microbial community varied between projects (crashed and non-crashed), feeding status (fed vs not), and strain (wild vs not). These data will be valuable to anyone interested in the microbiota of larval oysters, especially anyone interested in exploring hatchery crashes, effects of starvation, or strain level differences. They also contain a reproducible pipeline of amplicon analysis of host associated microbiota which may serve as a template for other studies. These data are a co-submission to a manuscript submitted to *Aquaculture* by Matthew grey et al. (2021) entitled Hatchery crashes among shellfish research hatcheries along the Atlantic coast of the United States: a case study at Horn Point Laboratory oyster hatchery. (Manuscript#: AQUACULTURE-D-21–01351R1)

## Specifications Table

**Table:** 

Subject	Microbiology: Microbiome
Specific subject area	Oyster hatchery larval microbiota
Type of data	Raw amplicon sequence data Amplicon sequence data pipeline Processed amplicon sequence data Analytical pipeline
How data were acquired	Data were acquired using an Illumina MiSeq
Data format	Raw Analysed Filtered
Parameters for data collection	Larvae were randomly sampled by filtration at one time-point during the experiment. Larvae were 7 (crash) and 5 (non-crash) days old at the “pre” time point. During the Starved and Fed time-points in the crash group they were 12 days old (5 days of starvation). During the Fed and Starved non-crash time-points they were 8 days old (3 days of starvation).
Description of data collection	Samples were extracted with SDS, heating and bead-beating, followed by proteinase K incubation. Samples were washed with phenol-chloroform three times, precipitated with isopropanol and ammonium acetate, rinsed with ethanol, and re-suspended in a Tris-EDTA solution. Amplicon library prep and sequencing was carried out by the Institute for Marine and Environmental Technologies' Bioanalytical Services Laboratory (IMET-BASLab). The Crashed samples, run after a laboratory shutdown following the 2020 pandemic, were initially of low quality and had to be re-run three additional times. The data presented here are from the best, fourth, run.
Data source location	Institution: University of Maryland, Center for Environmental Science – Horn Point Laboratory City/Town/Region: Cambridge, MD 21,613 Country: United States of America Latitude and longitude for collected samples/data: 38.585°N 76.137°W
Data accessibility	On public repositories Repository name: Sequence Read Archive Data identification number: PRJNA744562 Direct URL to data: https://www.ncbi.nlm.nih.gov/bioproject/PRJNA744562 Repository name: FigShare Data identification number: 15,025,446 Direct URL to data: https://figshare.com/articles/software/HPLOH_Crash/15025446 Instructions for accessing these data: SRA data can be downloaded following instructions on the SRA page, or using scripts in the FigShare repository. FigShare data can be downloaded directly or pulled from the mirrored GitHub repository.
Related research article	Matthew W. Gray, Stephanie Alexander, Brian Beal, Tom Bliss, Colleen A. Burge, Jacob Cram, Michael De Luca, James Dumhart, Patricia M. Glibert, Michael Gonsior, Andrew Heyes, Klaus Huebert, Vyacheslav Lyubchich, Matt Parker, Louis Plough, Eric Schott, Lisa Wainger, Ami Wilbur. In Press (2021). Hatchery crashes among shellfish research hatcheries along the Atlantic coast of the United States: a case study at Horn Point Laboratory oyster hatchery. Aquaculture (Manuscript#: AQUACULTURE-D-21–01351R1)

## Value of the Data

• These data show larval microbiota that are found associated with larval hosts with different health status, strain, and feeding status.
• Researchers who require raw or amplicon sequence data of oyster larval microbiota for analyses, or who would like to use any aspect of our bioinformatics and data processing pipeline, may want to use these data.
• Sequences were produced using commonly used universal primers and so can be merged with any dataset that use these primers. They will especially be valuable in larger analyses of oyster larval microbiota.

## 1 Data Description

**Dataset 1** are the raw sequence read files, and their metadata which are available on sequence read archive.

Sample names all follow the formatHPLOH_Project_Strain_Treatment_Replicate_Run_ReadDir.fastq.gz

**HPLOH**: Is the location, which is the same for all groups. The Horn Point Laboratory Oyster Hatchery.

**Project**: There were two projects, *Crash* one in which the larvae crashed (Started 27 June 2018) and *NoCrash* in which they did not crash (Started 14 August 2018). Also listed under “Project” are *Mock* communities made of compositions of sequences of known bacteria. They correspond to BEI Resources Low Concentration Mock communities. *Blank*, correspond to DNA extraction blanks. In extraction blanks, the DNA extraction process was applied in the absence of any larvae.

**Strain**: Both studies included a *Wild* type oyster, whose parents were harvested from the Chesapeake Bay, and a selectively bred strain. In the Crash experiment, this strain was *NEH* and in the NonCrash experiment it was *Aqua/LOLA*. Both strains are bred for disease resistance, while LOLA is also bred for low salinity tolerance. One group of oyster larvae, which were sampled during an early time-point for the Non-Crash experiment, were not properly recorded and so are listed as *Unknown*. Also found in the Strain column, corresponding to the Mock communities are an *Even* community made up of six species of the same DNA concentrations (Corresponding to BEI Resources Catalog No. HM-782D), and a *Staggered* community made of the same organisms, but in uneven abundances (HM-783D). *Blank* in this column, also refers to DNA extraction blanks.

**Treatment**: The experiments were designed as starvation trials. The *Starved* groups were starved from the beginning of the experiment to the sampling time-point (5 and 3 days for the Crash and NoCrash projects, respectively. The *Fed* groups were fed a standard hatchery diet throughout the experiment. *Pre* indicates the time-point just before the starvation began.

**Replicate**: Groups are in triplicate and are labelled *A, B* or *C* indicating the three replicates for each strain and treatment. The first time-point, when available has not yet been split into replicates.

**Run**: Two mi-seq illumina runs were performed by the BioAnalytical Services Laboratory. *NoCrash* corresponds to the first run in which the NonCrash samples were sequenced. *Crash4* corresponds to a later run in which the Crash samples, and a few NonCrashed organisms were sequenced, for comparison purposes.

**ReadDir**: We performed paired end sequencing. *R1* corresponds to the forward read, while *R2* corresponds to the reverse read.

**.fastq.gz:** All files are zipped fastq files, which contain both sequence and read-quality information

An example file name isCrash_Wild_Fed_A_Crash4_R2.fastq.gz

In this example, we see the microbiota from the *Crash* experiment, with *Wild* type larvae, that were *Fed* the whole experiment. They are from replicate *A*. They were sequenced in the second, *Crash4* run. This is a reverse read file (*R2*).

**Dataset 2** is a FigShare repository. It contains processed ASV count tables, estimates of taxonomy, and sample metadata. Additionally, it contains the informatics pipeline used to create these tables. It also contains reproducible scripts which generate the figure used in the co-submitted manuscript.

**Key data files** in this repository are found in the ***UsefulData*** subdirectory. They include:

**ASVs.fa:** A fasta file containing full sequences for each amplicon sequence variant.
**ASVs_counts.tsv**: An ASV count table. Each row is a different ASV, whose sequence can be found in *ASVs.fa* and whose taxonomy can be found in *ASVs_taxonomy.tsv*. Each column is a different sample, whose identify corresponds to the scheme described under *Dataset 1.*
**Asv_seqs.csv**: The same information as *ASVs.fa*, but in a table, rather than fasta file.
**ASVs_taxonomy**: A taxonomy table. Each row is an ASV, corresponding to sequences in *ASVs.fa*. Each column is a taxonomic level, between Kingdom and Genus. Taxonomies are based on the Silva classification scheme.
**Sample_data.csv**: Information about each sample. Columns correspond to the different groups described for Dataset 1. Rows correspond to each sample.

The analysis pipeline is saved in two R Markdown Notebooks:

**InitialProcessing.Rmd** which performs upstream DADA2 based calling of amplicon sequence variants, data cleaning and taxonomic classification.
**SecondaryProcessing.Rmd** performs the data analysis used in the main manuscript.
**README.md** provides an overview of the project. **Renv.lock** provides information about package versions used in all of the scripts.

Other directories contain the following information

***FigureEdits***: Figure files, edited in inkscape

***Figures***: Figure images, exported from R.

***Filtered, Renamed, Trimmed***: Empty directories, into which files are placed during processing.

***Scripts*:** Scripts. R scripts called by the analysis pipeline.

***PersonalLibraries***: Some R functions that I wrote that are used by the scripts.

***StartingData***: Data files used in the analysis pipeline. They are similar to the Key data files.

***IntermediateData***: Data files generated by the pipeline that are used in later steps.

***renv***: Mostly empty folder for recreating the renv environment so that package versions are the same when users run this as when I do.

## 2 Experimental Design, Materials and Methods

Larvae were collected from two experiments that were initially designed to explore the effects of starvation on oyster larval physiology and transcriptomics. However in this experiment, we looked instead at the microbiota associated with archived samples from that project. In each experiment newly spawned larvae were incubated in twelve 20 L lidded buckets in initially 1 µm carbon filtered seawater and fed a 50:50 diet of *Isochrysis galbana* and *Chaetoceros calcitrans*.

In the first experiment (hereafter known as the Crash project), initialized on June 27, 2018, two strains of larvae, wild type and the selectively bred NEH line, aged seven days, were obtained. The larvae were divided into twelve buckets, six per group, with each of those sub-divided into two treatments. Triplicate samples of each strain of larvae, were collected and stored in RNA later at −80 °C. The “Fed” control was fed daily for the next five days according the HPLOH standard hatchery protocol (Starting at 50,000 cell / mL (*t* = 0) and increased by 10,000 cells /mL each day thereafter) using a mixed species diet of *Isochrysis galbana* and *Chaetoceros*, while the “Starved” group was not fed over that period. After the five day period, another set of samples were archived. Over the next four days, all larvae in all groups died of an unknown cause, coincident with a die-off of all larvae in the adjoining oyster hatchery. During the experimental starvation period, mortality in the fed controls was high compared to the starved treatments, and observations during live survival counts showed decreasing gut coloration, suggesting that the fed controls were not feeding on the phytoplankton provided.

In the second experiment (hereafter the Non-Crash project), initialized on August 14, 2018, wild type larvae and larvae of the LOLA line, selectively bred for low salinity tolerance, aged five days were obtained. Again, the larvae were divided into twelve buckets, with half of the buckets starved and half not starved. Larvae were sampled after three days, and stored dry (no RNA later) at −80 C. This project continued for several more days (through settlement), with no crash. However, no further microbial samples were processed in this study.

These two experiments yielded 31 samples. Triplicates each, of fed and starved larvae, from two strains, from two projects for a total of 24 samples. Plus, an additional six samples, three from each strain before the start of the first experiment.

DNA was extracted from all samples using an in-house phenol chloroform extraction protocol. 400 µl of an SDS lysis buffer (1% SDS, containing 10 mM Tris-Cl (ph 8), and 1 mM EDTA) were added to each sample along with ∼100 µl of ultra-violet sterilized 1 mm diameter silica beads. Samples were incubated for two minutes at 95 °C, cooled on ice for three minutes, and then placed in a bead beater for 30 s. The process of heating for two minutes, cooling for three, and bead beating for 30 s was repeated two more times. Finally, 0.2 mg of proteinase K (VWR 39,450–01–6) was added and the samples were incubated at 57 °C for one hour. Samples were washed by adding 100 µl of Phenol-Chloroform-Isoamyl Alcohol solution (25:24:1, pH 8) and mixed by gentle inversion. The phenol layer was separated by centrifugation at 15,000 g and the organic phase was removed. A second wash with Phenol-Chloroform-Isoamyl Alcohol solution was performed, under the same conditions, followed with a final wash of Chloroform-Isoamyl Alcohol solution (24:1). 10 µg of Glycogen was added as a co-precipitate. DNA was precipitated by adding Ammonium acetate so that the final concentration was 2.5 M ammonium acetate and ethanol so that the final concentration of ethanol was 70%. Samples were mixed by inversion and precepted overnight (∼16 h) at 20 °C. DNA was extracted by spinning at 15,000 g for 60 min. The supernatant was removed, and the DNA pellet rinsed with 250 ml of 70% Ethanol, followed by another 30 min centrifugation. The pellet was dried by inverting the tubes and leaving them open for two hours. The pellet was then resuspended in 25µl of TE (10 mM Tris-Cl, 1 mM EDTA, pH8). Samples were quantified by Qubit fluorometer, following the manufacturer's protocol.

Samples were diluted to 5 ng/µl and transferred to Bioanalytical Services Lab at IMET for subsequent processing. Samples were run in two batches with samples from the Non-Crash project run first, and samples from the Crash project run second. Samples were processed following the 16S Metagenomic Sequencing Library Preparation Guide (Illumina, accessed 2019). Samples were amplified, following the manufacturer's protocol, using 515F-926R primers [1]. Samples were observed on an agarose gel, cleaned using Ampure beads, and then a second round of PCR was performed with Illumina indexing primers. Samples were again cleaned, validated, quantified, diluted to a final concentration of 4 nM. Samples were denatured, padded with PhiX, and loaded onto an Illumina MiSeq. Samples were automatically de-multiplexed using proprietary Illumina software and transferred back to the authors for subsequent analysis.

Fastq files were processed for the original manuscript following a protocol that was reproduced and released with this data-in brief. All steps of this analysis can be found in the FigShare repository, and broadly follow M. Lee's tutorial [2]. For that reproduction, files were uploaded to sequence read archive as Bioproject PRJNA744562. Scripts for downloading samples from SRA are available in the FigShare repository, indicated above. Samples were downloaded, renamed, and processed using the dada2 package [3] in R [4]. Primers were removed from samples with the cutadapt program in python [5]. Error rates were visualized and found to be high for the second “Crash” project run, so samples were run on the MiSeq three additional times, and re-processed. Only results from the fourth, and final run, which were of higher quality than the other two runs are shown here. Samples were filtered keeping only samples at least 175 bp long, and truncating samples to 230 bp for forward reads and 220 bp for reverse reads. Errors rates were learned separately for samples from the first (Non-Crash) and second (Crash) project. Samples were then dereplicated and amplicon sequence variants were called separately for each project. Or data were a mix of 16 and 18 s rRNA gene data and the 18 s reads were long enough that the forward and reverse reads did not overlap. Therefore, the 16 s reads were merged, while the 18 s reads were concatenated, and the 16 and 18 s datasets were combined using a custom script. The data from the Non-crash and Crash projects were then merged resulting in one common sequence table, containing ASV sequences and their relative abundances for the entire shared project. Chimeras were removed, and taxonomy was assigned to each sequence. Finally taxonomy tables, ASV sequences, count data, and metadata were merged into a phyloseq object [6].

Count data were normalized using a variance stabilizing transformation using the deseq2 package in R [7]. This allows us to correct for differences in sequencing depth without rarefying the data. The total number of counts in each sample corresponding to ASVs that matched oyster 18 s sequences was calculated. Data were transformed by dividing the counts of each non-oyster ASV by the total oyster ASV, resulting in a ratio of microbial ASV to host gene copy number. This transformation allowed us to avoid compositionality problems characteristic of relative abundance data. Furthermore, it provided us with a metric that was proportional to how abundant microbes were relative to host biomass. Redundancy analysis was performed with the vegan package [8] to explore the interaction between community composition and the interactions between project (Crash vs Non-Crash), feeding status (Fed vs Starved) and strain (Wild vs Low salinity tolerant). This figure is presented in the companion paper. Furthermore, linear mixed effects models were used to compare the log transformed abundance ratios of each species against the project (Crash vs Non-Crash) accounting for differences between treatments and strains using the lme4 package [9]. All analyses described herein are presented in the FigShare repository and connected GitHub repository. All results from this analysis are presented in the companion paper [10].

## Ethics Statement

All data herein are original to the joint submission of this and its companion paper. Data are represented accurately to the knowledge of the authors. All data are publicly available and can be accessed as described in this manuscript. This is an entirely original work, though some language found herein is also used in the public data repositories. All authors contributed to this work. No vertebrate animals were used, and all work with animals (oyster larvae) are exempt from NIH guidelines for the care and use of laboratory animals. However, all efforts at humane care of the larvae were applied. No human subjects or social media data were collected in this project.

## CRediT authorship contribution statement

**Jacob A. Cram:** Conceptualization, Methodology, Supervision, Software, Visualization, Validation, Writing – original draft. **Matthew W. Gray:** Supervision, Writing – review & editing. **Katherine McFarland:** Conceptualization, Methodology, Writing – review & editing. **Ashley Hollins:** Investigation.

## Declaration of Competing Interest

The authors declare that they have no known competing financial interests or personal relationships which have or could be perceived to have influenced the work reported in this article.
